# Deletions at 14q in malignant mesothelioma detected by microsatellite marker analysis

**DOI:** 10.1038/sj.bjc.6690816

**Published:** 1999-12

**Authors:** A-M Björkqvist, M Wolf, S Nordling, L Tammilehto, A Knuuttila, J Kere, K Mattson, S Knuutila

**Affiliations:** Departments of Medical Genetics andPathology, Haartman Institute, PO Box 21, FIN-00014 Helsinki,University of Helsinki, Helsinki, Finland; Laboratory of Medical Genetics andDepartment of Medicine, Division of Pulmonary Medicine, Helsinki University Central Hospital, Helsinki, Finland; Department of Epidemiology and Biostatistics, Finnish Institute of Occupational Health, Helsinki, Finland; Finnish Genome Center, University of Helsinki, Helsinki, Finland

**Keywords:** microsatellite marker analysis, loss of heterozygosity, allelic imbalance, malignant mesothelioma

## Abstract

Previous molecular cytogenetic studies by comparative genomic hybridization (CGH) on primary tumours of human malignant mesothelioma have revealed that loss of genetic material at chromosome 14q is one of the most frequently occurring aberrations. Here we further verify the frequency and pattern of deletions at 14q in mesothelioma. A high-resolution deletion mapping analysis of 23 microsatellite markers was performed on 18 primary mesothelioma tumours. Eight of these had previously been analysed by CGH. Loss of heterozygosity or allelic imbalance with at least one marker was detected in ten of 18 tumours (56%). Partial deletions of varying lengths were more common than loss of all informative markers, which occurred in only one tumour. The highest number of tumours with deletions at a specific marker was detected at 14q11.1–q12 with markers D14S283 (five tumours), D14S972 (seven tumours) and D14S64 (five tumours) and at 14q23–q24 with markers D14S258 (five tumours), D14S77 (five tumours) and D14S284 (six tumours). We conclude from these data that genomic deletions at 14q are more common than previously reported in mesothelioma. Furthermore, confirmation of previous CGH results was obtained in all tumours but one. This tumour showed deletions by allelotyping, but did not show any DNA copy number change at 14q by CGH. Although the number of tumours allelotyped was small and the deletion pattern was complex, 14q11.1–q12 and 14q23–q24 were found to be the most involved regions in deletions. These regions provide a good basis for further molecular analyses and may highlight chromosomal locations of tumour suppressor genes that could be important in the tumorigenesis of malignant mesothelioma. © 1999 Cancer Research Campaign


*British Journal of Cancer* (1999) **81**(7), 1111–1115
© 1999 Cancer Research Campaign
Article no. bjoc.1999.0816

### Deletions at 14q in malignant mesothelioma detected by microsatellite marker analysis

**A-M Björkqvist[1], M Wolf[1,3], S Nordling[2], L Tammilehto[5], A Knuuttila[4], J Kere[6], K Mattson[4] and S Knuutila[1,3]**

Departments of [1]Medical Genetics and [2]Pathology, Haartman Institute, PO Box 21, FIN-00014, University of Helsinki, Helsinki, Finland; [3]Laboratory of Medical Genetics and [4]Department of Medicine, Division of Pulmonary Medicine, Helsinki University Central Hospital, Helsinki, Finland; [5]Department of Epidemiology and Biostatistics, Finnish Institute of Occupational Health, Helsinki, Finland; [6]Finnish Genome Center, University of Helsinki, Helsinki, Finland

**Summary** Previous molecular cytogenetic studies by comparative genomic hybridization (CGH) on primary tumours of human malignant mesothelioma have revealed that loss of genetic material at chromosome 14q is one of the most frequently occurring aberrations. Here we further verify the frequency and pattern of deletions at 14q in mesothelioma. A high-resolution deletion mapping analysis of 23 microsatellite markers was performed on 18 primary mesothelioma tumours. Eight of these had previously been analysed by CGH. Loss of heterozygosity or allelic imbalance with at least one marker was detected in ten of 18 tumours (56%). Partial deletions of varying lengths were more common than loss of all informative markers, which occurred in only one tumour. The highest number of tumours with deletions at a specific marker was detected at 14q11.1–q12 with markers D14S283 (five tumours), D14S972 (seven tumours) and D14S64 (five tumours) and at 14q23–q24 with markers D14S258 (five tumours), D14S77 (five tumours) and D14S284 (six tumours). We conclude from these data that genomic deletions at 14q are more common than previously reported in mesothelioma. Furthermore, confirmation of previous CGH results was obtained in all tumours but one. This tumour showed deletions by allelotyping, but did not show any DNA copy number change at 14q by CGH. Although the number of tumours allelotyped was small and the deletion pattern was complex, 14q11.1–q12 and 14q23–q24 were found to be the most involved regions in deletions. These regions provide a good basis for further molecular analyses and may highlight chromosomal locations of tumour suppressor genes that could be important in the tumorigenesis of malignant mesothelioma.
© 1999 Cancer Research Campaign

**Keywords:** microsatellite marker analysis; loss of heterozygosity; allelic imbalance; malignant mesothelioma

Malignant mesothelioma, a tumour frequently linked to past occupational exposure to asbestos, arises from mesothelial cells of the pleura, peritoneum or pericardium (Wagner et al, 1960). Mesothelioma is characterized by a long latent period (35–40 years) (Browne, 1986; Yates et al, 1997) and due to the lack of efficient treatment, its prognosis is very poor. From 1975 to 1990 there was an increasing trend in the incidence of mesotheliomas in Finland, but since 1990 it has slowed down. Based on observations from 1985 to 1995, by 2010 the annual incidence of mesotheliomas among men and women is expected to be around 40–50 and 10–20 cases respectively (Karjalainen et al, 1997).

Previous cytogenetic and molecular genetic studies have indicated several chromosomal locations with frequent alterations in mesothelioma (Tiainen et al, 1989; Hagemeijer et al, 1990; Taguchi et al, 1993; Cheng et al, 1994; Lu et al, 1994; Zeiger et al, 1994; Bianchi et al, 1995; Sekido et al, 1995). However, no aberration specific to mesothelioma has been detected. Comparative genomic hybridization (CGH) analyses on primary mesothelioma tumours and cell lines have revealed that loss of genetic material at chromosome 14q is one of the most frequently occurring aberrations (Kivipensas et al, 1996; Björkqvist et al, 1997, 1998; Balsara et al, 1999). In this study we further verify the frequency and pattern of deletions at 14q in primary mesothelioma tumours using polymerase chain reaction (PCR) and 23 microsatellite markers spanning the whole of the long arm of chromosome 14.

*Received 15 February 1999*
*Revised 21 April 1999*
*Accepted 13 May 1999*

*Correspondence to:* S Knuutila, Laboratory of Medical Genetics, Helsinki University Central Hospital, PO Box 404 (Haartmaninkatu 3, 4th floor), FIN-00029 HUCH, Helsinki, Finland

### MATERIALS AND METHODS

### Tumours and clinical characteristics of patients

Eighteen primary tumours of patients with malignant mesothelioma were included in the microsatellite marker analysis. All of the patients had undergone surgery at the Helsinki University Central Hospital during years 1995–1997. Three of the patients were females and 15 males. The mean age at diagnosis was 57 years (range 44–72 years). Thirteen patients had a clear history of asbestos exposure. Three tumour samples were from patients with stage 2 disease, whereas eight and seven samples were from patients with stage 3 and 4 disease respectively. The median survival of the patients was 16 months. The histological classification of the tumours was performed by the Finnish Mesothelioma Panel. Fourteen tumours were epithelial (nos 1–14), two mixed (nos 15 and 16), one fibromatous (no. 17) and in one case the histological subtype could not be determined (no. 18). CGH analyses had previously been performed on eight of the tumours (Table 1).

Tumour DNA was extracted from paraffin-embedded tissues (nos 1–13 and 15–18) by a salting out procedure (Miller et al, 1988) or from fresh-frozen material (no. 14) according to standard protocols. The proportion of malignant cells in the tumour samples

**Table 1** Pattern and frequency of loss of heterozygosity (LOH) and allelic imbalance (AI) detected with 23 microsatellite markers at 14q in 18 primary mesothelioma tumours

| | Tumour[a] | | | | | | | | | | | | | | | | | | | | |
|---|---|---|---|---|---|---|---|---|---|---|---|---|---|---|---|---|---|---|---|---|---|
| Marker[b] | 1[c] | 2[d] | 3[e] | 4[f] | 5 | 6 | 7 | 8 | 9 | 10[g] | 11 | 12 | 13[h] | 14[i] | 15[j] | 16 | 17 | 18 | Tumours with LOH or AI/total informative | LOH or AI (%) | cM[k] |
| D14S72 | ■ | ■ | ■ | □ | □ | □ | — | ND | X | ND | □ | □ | □ | □ | □ | □ | □ | ■ | 4/14 | 29 | 1.4 |
| D14S1003 | ◨ | — | □ | — | □ | ■ | □ | — | — | □ | □ | □ | □ | — | □ | — | □ | — | 2/11 | 18 | 4.0 |
| D14S283 | ■ | ■ | ■ | ◨ | □ | □ | □ | — | — | □ | □ | □ | □ | □ | □ | □ | □ | ■ | 5/16 | 31 | 7.6 |
| D14S972 | ■ | ◨ | ◨ | □ | ◨ | ◨ | □ | X | □ | ND | □ | □ | □ | X | ◨ | □ | X | ■ | 7/14 | 50 | 1.2 |
| D14S64 | ■ | ◨ | ■ | □ | □ | — | — | ◨ | ND | ND | — | □ | □ | □ | ND | □ | — | ■ | 5/11 | 45 | 4.3 |
| D14S80 | □ | X | □ | — | ◨ | — | — | □ | □ | □ | — | □ | □ | □ | — | □ | X | ■ | 2/11 | 18 | 7.4 |
| D14S1060 | □ | — | — | — | □ | □ | □ | □ | — | □ | □ | □ | □ | □ | □ | □ | □ | ■ | 1/14 | 7 | 8.3 |
| D14S75 | ■ | ■ | — | — | □ | □ | — | □ | — | □ | □ | □ | — | □ | ■ | □ | — | ■ | 4/12 | 33 | 6.6 |
| D14S269 | ■ | ■ | ND | — | — | □ | ND | X | X | □ | □ | □ | □ | X | — | □ | X | ■ | 3/9 | 33 | 7.1 |
| D14S66 | ■ | — | □ | ◨ | □ | □ | □ | ◨ | □ | — | □ | □ | □ | □ | □ | — | □ | — | 3/14 | 21 | 9.0 |
| D14S63 | ■ | □ | □ | ◨ | □ | □ | □ | ◨ | X | □ | □ | □ | □ | □ | □ | □ | X | ■ | 4/16 | 25 | 6.8 |
| D14S258 | ■ | — | ■ | □ | — | □ | ■ | ◨ | X | — | — | □ | ND | □ | ■ | □ | □ | — | 5/11 | 45 | 2.2 |
| D14S277 | — | X | ◨ | — | — | □ | □ | X | □ | — | □ | □ | — | □ | X | □ | X | — | 1/8 | 13 | 1.8 |
| D14S77 | □ | □ | ■ | ◨ | ■ | □ | □ | □ | X | □ | □ | □ | □ | □ | ■ | □ | ND | ■ | 5/16 | 31 | 1.7 |
| D14S71 | X | X | □ | □ | □ | — | — | X | □ | — | □ | □ | □ | □ | ND | □ | X | — | 0/9 | 0 | 1.7 |
| D14S284 | ■ | □ | ■ | — | — | ■ | ◨ | ◨ | — | □ | — | — | — | □ | ◨ | — | □ | — | 6/10 | 60 | 0.5 |
| D14S76 | — | □ | — | — | ◨ | □ | — | ◨ | — | □ | — | □ | □ | □ | ◨ | □ | □ | — | 3/11 | 27 | 2.7 |
| D14S74 | — | □ | ◨ | □ | □ | □ | — | ◨ | X | □ | — | — | — | □ | — | □ | — | ■ | 3/10 | 30 | 9.9 |
| D14S68 | ■ | ND | □ | □ | ■ | ■ | □ | □ | □ | — | □ | □ | □ | — | — | □ | ND | ■ | 4/13 | 31 | 12.5 |
| D14S81 | ■ | ND | □ | — | □ | □ | ■ | ND | □ | ND | □ | □ | ND | □ | □ | □ | □ | ■ | 3/13 | 23 | 9.3 |
| D14S65 | ■ | □ | □ | □ | □ | □ | □ | ◨ | □ | — | □ | □ | □ | □ | □ | — | □ | ND | 2/15 | 13 | 8.2 |
| D14S78 | ■ | □ | □ | — | □ | □ | □ | □ | □ | □ | □ | □ | □ | □ | ND | □ | — | ■ | 2/15 | 13 | 12.3 |
| D14S1007 | — | □ | ■ | — | □ | — | □ | X | □ | — | — | □ | □ | □ | □ | □ | — | ■ | 2/11 | 18 | 0.0 |

[a]Tumours 1–14 are epithelial, 15 and 16 mixed, 17 fibromatous and 18 could not be determined; [b]microsatellite markers are placed in the predicted order from cen-qter; [c,d,e,f,g,h]cases 9, 21, 23, 11, 20 and 6 in Björkqvist et al (1998) respectively; [i,j]cases 4 and 14 in Björkqvist et al (1997) respectively; [k]genetic distance between markers in cM; □ = Retained heterozygosity; ■ = LOH; ◨ = AI; — = Uninformative; ND = Not determined; X = no data was at least 60%. Normal DNA from each patient was isolated from peripheral blood samples according to standard methods.

### Microsatellite marker analysis

A total of 23 dinucleotide microsatellite markers were used in the analysis. The samples were first screened using 15 microsatellite markers: D14S72, D14S283, D14S80, D14S1060, D14S75, D14S269, D14S66, D14S63, D14S77, D14S74, D14S68, D14S81, D14S65, D14S78 and D14S1007. These spanned the whole q-arm with an average genetic distance of 9 cM (range 5.4–13.1 cM) between the markers. To further refine the locations with the highest number of tumours with loss of heterozygosity (LOH) or allelic imbalance (AI), eight additional microsatellite markers (D14S1003, D14S972, D14S64, D14S258, D14S277, D14S71, D14S284 and D14S76) were analysed. The relative order of markers and genetic distances between them were obtained from the Généthon genetic linkage map (http: //www.genethon.fr) (Dib et al, 1996). Cytogenetic locations of the markers were based on the Genome Database (The Johns Hopkins University BioInformatics Web Server) at http: //www.bis.med.jhmi.edu/.

The PCR reactions were carried out in an MJ Research PTC-200 Peltier Thermal Cycler. The reaction was done in a 20 μl volume containing 50 ng DNA, 10 mM Tris–HCl (pH 8.3), 50 mM potassium chloride, 1.5 mM magnesium chloride, 0.001% gelatin (w/v), 1% dimethyl sulphoxide (w/v), 0.5 μM of each primer, 200 μM each of dATP, dCTP, dGTP and dTTP, and 0.6 unit of *AmpliTaq* DNA Polymerase (Perkin-Elmer, Branchburg, NJ, USA). The following amplification programme was used: an initial denaturation at 94°C for 4 min followed by 28 cycles of 30 s at 94°C, 1 min 15 s at 55°C, and 15 s at 72°C and then by a 6-min extension at 72°C. The annealing temperature for different microsatellite markers varied between 55°C and 65°C. For some of the samples the extension time was increased from 15 to 45 s. The amplification product was diluted 1:1 in a 95% formamide gel-loading buffer, denatured at 93°C for 3 min, after which 5 μl was loaded on a 6% denaturing polyacrylamide sequencing gel (National Diagnostics Inc., Atlanta, GA, USA). After the gel was fixed in 10% citric acid, the PCR products were visualized by silver staining. The gels were scanned using a UMAX Power Look 2000 (UMAX Data Systems Inc., Hsinchu, Taiwan).

The analysis was done visually and independently by two observers by comparing the intensities between the alleles in tumour DNA to those in normal DNA. Tumours homozygous for a specific locus were defined as uninformative. LOH was observed in heterozygous samples when one of the alleles in the tumour

*© 1999 Cancer Research Campaign*

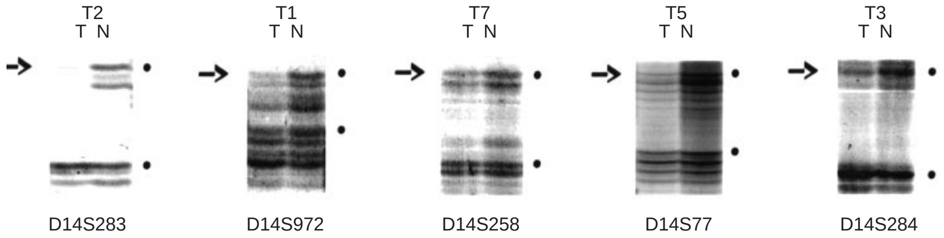

**Figure 1** Representative results of microsatellite markers D14S283, D14S972, D14S258, D14S77, and D14S284. Tumour numbers are shown at top. Locations of alleles are indicated with a dot. Arrowheads, LOH. Faint signals in the tumour lanes may result from normal cell contamination or intratumour heterogeneity. T, primary tumour DNA; N, normal DNA from peripheral blood

DNA was reduced in intensity relative to the remaining allele and the alleles in the normal DNA. Other intensity differences were interpreted merely as AIs. All reactions displaying LOH or AI were repeated. If the allele pattern was interpreted as LOH in one experiment and as AI in the other, it was considered to be an AI. However, the reason some of the observed AIs were not determined as LOHs could be that tumour DNA was amplified more efficiently than normal DNA. Moreover, previous CGH data of, for instance, tumour no. 4 confirm the presence of loss of DNA sequences at 14q, although allelotyping was interpreted as AI. Therefore we refer to deletion for both LOH and AI.

### RESULTS

Ten of 18 (56%) mesothelioma tumours displayed LOH or allelic imbalance with at least one microsatellite marker at 14q (Table 1). Eight of these were epithelial tumours, one a mixed tumour and one a tumour with undetermined histology. Partial deletions of varying lengths, detected as LOH or AI of some loci and as retention of heterozygosity of others, were more common than loss of the entire q-arm. Deletions of all informative markers, revealing deletion of the whole long arm which indicates monosomy 14, were found in only one tumour (no. 18). In four tumours deletions of several adjacent markers were observed, whereas the remaining five tumours displayed deletions of two adjacent markers or only at one marker. In some of these the marker that showed deletion was, however, flanked by an uninformative marker or a marker, whose status could not be determined.

Based on the results of the first 15 microsatellite markers analysed, the highest number of tumours carrying a deletion was found for markers D14S283 (five tumours) and D14S77 (five tumours) (Table 1). The density of mapping was increased around these markers by analysing additional eight markers. Based on the results from all 23 markers, 14q11.1–q12 and 14q23–q24 were found to be the most involved regions in deletions. Representative results of some markers are presented in Figure 1. The highest number of tumours displaying deletions at 14q11.1–q12 was detected with markers D14S283 (five tumours; frequency: 31%), D14S972 (seven tumours; frequency: 50%) and D14S64 (five tumours; frequency: 45%). Four tumours had deletions with all three markers. The highest number of tumours displaying deletions at 14q23–q24 was observed with markers D14S258 (five tumours; frequency: 45%), D14S77 (five tumours; frequency: 31%) and D14S284 (six tumours; frequency: 60%). Only one tumour (frequency: 13%) displayed a deletion with marker D14S277 (between D14S258 and D14S77) and no deletions were detected with marker D14S71 (between D14S77 and D14S284; Table 1).

Eight tumours displayed deletions at both 14q11.1–q12 and 14q23–q24 with at least one marker. Tumour no. 2, which displayed deletions only in the proximal part of 14q, was uninformative at D14S258 but contained both alleles at loci detected with markers D14S77 and D14S284. Tumour no. 7, which displayed deletion at 14q23–q24 but not at 14q11.1–q12, was uninformative at D14S64 but displayed retention of heterozygosity at both D14S283 and D14S972 (Table 1).

Patients, in whose tumour DNA we detected deletions at 14q, had a shorter median survival (12 months) than those without deletions at 14q in their tumours (20 months). Five of the eight patients with no detectable deletion at 14q in their tumours survived more than 1.5 years compared to only two of the ten with a deletion at 14q in their tumours. However, the difference in survival between these two groups, analysed using log-rank and Wilcoxon tests, was not statistically significant. Nine out of 11 patients with stage 2 or 3 disease had deletions at 14q in their tumours, as compared to only one of the seven patients with stage 4 disease ($P = 0.013$, Fisher's exact test, two-tailed). In nine of the 13 tumours from patientswith a clear history of asbestos exposure, deletions at 14q were detected, as compared to only one of the five patients with no known exposure to asbestos (not statistically significant). The small number of samples analysed does not allow decisive conclusions to be drawn from the statistical analyses.

### DISCUSSION

Eighteen primary malignant mesothelioma tumours were analysed using 23 microsatellite markers spanning the whole long arm of chromosome 14 in order to characterize the frequency and pattern of deletions at 14q. Fifty-six per cent of the tumours displayed a deletion. The percentage is much higher than that (24%) observed in primary tumours by CGH (Kivipensas et al, 1996; Björkqvist et al, 1997, 1998). However, this is not unexpected due to the small partial deletions observed by allelotyping in some of the tumours and because of the resolution limitation of the CGH method (Kallioniemi et al, 1994). Recent allelotyping analyses at 1p and 6q on primary mesothelioma tumours have revealed frequencies of 61% (11/18) and 38% (6/16) for allelic loss respectively (Lee et al, 1996; Bell et al, 1997).

In eight of the tumours (nos 1, 2, 3, 4, 10, 13, 14 and 15) we were able to compare the allelotyping results with previous CGH data of 14q (Björkqvist et al, 1997, 1998). Loss of genetic material at 14q was found by CGH in tumour nos 1, 3, 4 and 15. In all of these we found deletions for several markers. Tumour nos 10, 13 and 14, which did not show any loss of DNA sequences at 14q by CGH, did not display any deletion by allelotyping either. However, tumour no. 2, whose CGH profile of 14q did not exceed the

© 1999 Cancer Research Campaign threshold for loss of genetic material, displayed deletions at the proximal part of 14q by allelotyping.

In earlier cytogenetic analyses deletions at chromosome 14 have not been among the most frequently observed chromosomal aberrations in mesothelioma. However, monosomy 14 has been detected (Tiainen et al, 1989; Taguchi et al, 1993). The deletion pattern that we observed in the present allelotyping analysis was very complex. Only one of the tumours displayed deletion of all informative markers revealing a loss of the whole chromosome. Partial deletions of varying lengths were more common, suggesting a complex mechanism, which perhaps involves both chromosomal deletions and rearrangements, for inactivation of tumour suppressor genes. Such complexity at the chromosomal level was evident in a recent mesothelioma tumour analysed in our diagnostic laboratory (data not shown). By G-banding a chaotic karyotype was observed with a hypodiploid modal chromosome number and seven unidentified marker chromosomes. Both copies of chromosome 14 were missing. Chromosome painting analysis using a chromosome 14-specific library probe revealed one of the smaller markers to be composed of material from chromosome 14 [del(14q)] and part of chromosome 14 to be translocated to two unidentified marker chromosomes. As a result there was a net loss of genetic material from chromosome 14. This loss was further mapped by CGH to 14qcen–q21. Taken together these results manifest the complexity and suggest possible impact of deletions at 14q in mesothelioma. Thus, it would appear that profound chromosomal instability accumulates aberrant cells, of which some exhibit genetic changes advantageous in tumorigenesis.

Although the deletion pattern in the present study was complex, and most probably induced by chromosomal aberrations, 14q11.1–q12 and 14q23–q24 were found to be the most involved regions in deletions. 14q11.1–q12 overlaps those regions that have been found deleted in ovarian carcinoma and bladder cancer (14q12–q13 and 14q12 respectively). However, the critical markers in these studies, in contrast to ours, were all located distal to D14S64 (Chang et al, 1995; Bandera et al, 1997). In lung carcinoma the highest frequency of LOH was found with marker D14S261 at 14q11.1–q11.2 (centromeric to D14S72) (Abujiang et al, 1998). We observed deletions at 14q23–q24 by microsatellite markers D14S258, D14S77 and D14S284 in five, five and six tumours respectively. Interestingly, Schwerdtle et al have found that marker D14S258 also exhibits frequent allelic loss in renal oncocytomas, implicating a locus for a tumour suppressor gene possibly involved in different tumours (Schwerdtle et al, 1997). Furthermore, 14q24–q32 has been found to be the smallest overlapping segment of deletions in both nonpapillary renal cell carcinoma and meningiomas (Simon et al, 1995; Herbers et al, 1997).

No tumour suppressor genes have so far been assigned to 14q. However, the *APEX* gene located at 14q11.2–q12 falls into one of the regions that we found to be most involved in deletions. This gene encodes a DNA repair enzyme called APEX nuclease or apurinic endonuclease (APE), which initiates the repair of abasic sites caused by mutagens such as free oxygen radicals (Demple et al, 1991; Harrison et al, 1992). Such oxygen radicals, with DNA-damaging properties, are known to be released by macrophages exposed to asbestos (Hansen and Mossman, 1987; Goodglick and Kane, 1990). Inactivation of enzymes involved in DNA repair, including *APEX*, would prevent their functions and leave the damaged DNA uncorrected, thereby causing mutations. The expression level of APEX in mesothelioma has, as far as we know, not been studied. However, increased production of APEX has been detected in rat pleural mesothelial cells, but not in Met5A cells (an immortalized human mesothelial cell line), as a response to asbestos exposure (Fung et al, 1998).

In conclusion, the present microsatellite marker analysis suggests that genomic losses at 14q are more frequent in mesothelioma than previously recognized by other methods. Partial deletions were more common than loss of the whole arm and sometimes included only a small chromosomal segment. Although the deletion pattern seems to be complex, deletion at both 14q11.1–q12 and 14q23–q24 was observed in eight of the ten tumours exhibiting losses. The two remaining tumours had deletion either at 14q11.1–q12 or 14q23–q24.

These chromosomal regions provide a good basis for further fine-mapping and can help in pinpointing the locations of genes, which may be important in the tumorigenesis of malignant mesothelioma.

### ACKNOWLEDGEMENTS

The authors thank Siv Knaappila, Tuula Niilola and Sari Rantanen for technical advice and assistance. The study was supported by the Helsinki University Central Hospital, the Finnish Cancer Society, the Ida Montin Foundation, the University of Helsinki and the Academy of Finland.

### REFERENCES

Abujiang P, Mori TJ, Takahashi T, Tanaka F, Kasyu I, Hitomi S and Hiai H (1998) Loss of heterozygosity (LOH) at 17q and 14q in human lung cancers. *Oncogene* **17**: 3029–3033

Balsara BR, Bell DW, Sonoda G, De Rienzo A, du Manoir S, Jhanwar SC and Testa JR (1999) Comparative genomic hybridization and loss of heterozygosity analyses identify a common region of deletion at 15q11.1–15 in human malignant mesothelioma. *Cancer Res* **59**: 450–454

Bandera CA, Takahashi H, Behbakht K, Liu PC, LiVolsi VA, Benjamin I, Morgan MA, King SA, Rubin SC and Boyd J (1997) Deletion mapping of two potential chromosome 14 tumor suppressor gene loci in ovarian carcinoma. *Cancer Res* **57**: 513–515

Bell DW, Jhanwar SC and Testa JR (1997) Multiple regions of allelic loss from chromosome arm 6q in malignant mesothelioma. *Cancer Res* **57**: 4057–4062

Bianchi AB, Mitsunaga S-I, Cheng JQ, Klein WM, Jhanwar SC, Seizinger B, Kley N, Klein-Szanto AJP and Testa JR (1995) High frequency of inactivating mutations in the neurofibromatosis type 2 gene (*NF2*) in primary malignant mesothelioma. *Proc Natl Acad Sci USA* **92**: 10854–10858

Björkqvist A-M, Tammilehto L, Anttila S, Mattson K and Knuutila S (1997) Recurrent DNA copy number changes in 1q, 4q, 6q, 9p, 13q, 14q and 22q detected by comparative genomic hybridization in malignant mesothelioma. *Br J Cancer* **75**: 523–527

Björkqvist A-M, Tammilehto L, Nordling S, Nurminen M, Anttila S, Mattson K and Knuutila S (1998) Comparison of DNA copy number changes in malignant mesothelioma, adenocarcinoma and large-cell anaplastic carcinoma of the lung. *Br J Cancer* **77**: 260–269

Browne K (1986) Mesothelioma registry data. *Lancet* ii(8499) 167

Chang WY-H, Cairns P, Schoenberg MP, Polascik TJ and Sidransky D (1995) Novel suppressor loci on chromosome 14q in primary bladder cancer. *Cancer Res* **55**: 3246–3249

Cheng JQ, Jhanwar SC, Klein WM, Bell DW, Lee W-C, Altomare DA, Nobori T, Olopade OI, Buckler AJ and Testa JR (1994) *p16* alterations and deletion mapping of 9p21–p22 in malignant mesothelioma. *Cancer Res* **54**: 5547–5551

Demple B, Herman T and Chen DS (1991) Cloning and expression of APE, the cDNA encoding the major human apurinic endonuclease: Definition of a family of DNA repair enzymes. *Proc Natl Acad Sci USA* **88**: 11450–11454

Dib C, Fauré S, Fizames C, Samson D, Drouot N, Vignal A, Millasseau P, Marc S, Hazan J, Seboun E, Lathrop M, Gyapay G, Morissette J and Weissenbach J (1996) A comprehensive genetic map of the human genome based on 5,264 microsatellites. *Nature* **380**: 152–154

Fung H, Kow YW, Van Houten B, Taatjes DJ, Hatahet Z, Janssen YMW, Vacek P, Faux SP and Mossman BT (1998) Asbestos increases mammalian

 *© 1999 Cancer Research Campaign*

AP-endonuclease gene expression, protein levels, and enzyme activity in mesothelial cells. *Cancer Res* **58**: 189–194

Goodglick LA and Kane AB (1990) Cytotoxicity of long and short crocidolite asbestos fibers in vitro and in vivo. *Cancer Res* **50**: 5153–5163

Hagemeijer A, Versnel MA, Van Drunen E, Moret M, Bouts MJ, van der Kwast TH and Hoogsteden HC (1990) Cytogenetic analysis of mesothelioma. *Cancer Genet Cytogenet* **47**: 1–28

Hansen K and Mossman BT (1987) Generation of superoxide from alveolar macrophages exposed to asbestiform and nonfibrous particles. *Cancer Res* **47**: 1681–1686

Harrison L, Ascione G, Menninger JC, Ward DC and Demple B (1992) Human apurinic endonuclease gene (*APE*): structure and genomic mapping (chromosome 14q11.2–12). *Hum Molec Genet* **1**: 677–680

Herbers J, Schullerus D, Muller H, Kenck C, Chudek J, Weimer J, Bugert P and Kovacs G (1997) Significance of chromosome arm 14q loss in nonpapillary renal cell carcinomas. *Genes Chromosomes Cancer* **19**: 29–35

Kallioniemi O-P, Kallioniemi A, Piper J, Isola J, Waldman FM, Gray JW and Pinkel D (1994) Optimizing comparative genomic hybridization for analysis of DNA sequence copy number changes in solid tumors. *Genes Chromosomes Cancer* **10**: 231–243

Karjalainen A, Pukkala E, Mattson K, Tammilehto L and Vainio H (1997) Trends in mesothelioma incidence and occupational mesotheliomas in Finland in 1960–1995. *Scand J Work Environ Health* **23**: 266–270

Kivipensas P, Björkqvist A-M, Karhu R, Pelin K, Linnainmaa K, Tammilehto L, Mattson K, Kallioniemi O-P and Knuutila S (1996) Gains and losses of DNA sequences in malignant mesothelioma by comparative genomic hybridization. *Cancer Genet Cytogenet* **89**: 7–13

Lee W-C, Balsara B, Liu Z, Jhanwar SC and Testa JR (1996) Loss of heterozygosity analysis defines a critical region in chromosome 1p22 commonly deleted in human malignant mesothelioma. *Cancer Res* **56**: 4297–4301

Lu YY, Jhanwar SC, Cheng JQ and Testa JR (1994) Deletion mapping of the short arm of chromosome 3 in human malignant mesothelioma. *Genes Chromosomes Cancer* **9**: 76–80

Miller SA, Dykes DD and Polesky HF (1988) A simple salting out procedure for extracting DNAs from human nucleated cells. *Nucleic Acids Res* **16**: 1215

Schwerdtle RF, Winterpacht A, Störkel S, Brenner W, Hohenfellner R, Zabel B, Huber C and Decker H-J (1997) Loss of heterozygosity studies and deletion mapping identify two putative chromosome 14q tumor suppressor loci in renal oncocytomas. *Cancer Res* **57**: 5009–5012

Sekido Y, Pass HI, Bader S, Mew DJY, Christman MF, Gazdar AF and Minna JD (1995) Neurofibromatosis type 2 (*NF2*) gene is somatically mutated in mesothelioma but not in lung cancer. *Cancer Res* **55**: 1227–1231

Simon M, von Deimling A, Larson JJ, Wellenreuther R, Kaskel P, Waha A, Warnick RE, Tew JM Jr and Menon AG (1995) Allelic losses on chromosomes 14, 10, and 1 in atypical and malignant meningiomas: A genetic model of meningioma progression. *Cancer Res* **55**: 4696–4701

Taguchi T, Jhanwar SC, Siegfried JM, Keller SM and Testa JR (1993) Recurrent deletions of specific chromosomal sites in 1p, 3p, 6q, and 9p in human malignant mesothelioma. *Cancer Res* **53**: 4349–4355

Tiainen M, Tammilehto L, Rautonen J, Tuomi T, Mattson K and Knuutila S (1989) Chromosomal abnormalities and their correlations with asbestos exposure and survival in patients with mesothelioma. *Br J Cancer* **60**: 618–626

Wagner JC, Sleggs CA and Marchand P (1960) Diffuse pleural mesotheliomas and asbestos exposure in the Northwestern Cape Province. *Br J Ind Med* **17**: 260–271

Yates DH, Corrin B, Stidolph PN and Browne K (1997) Malignant mesothelioma in south east England: clinicopathological experience of 272 cases. *Thorax* **52**: 507–512

Zeiger MA, Gnarra JR, Zbar B, Linehan WM and Pass HI (1994) Loss of heterozygosity on the short arm of chromosome 3 in mesothelioma cell lines and solid tumors. *Genes Chromosomes Cancer* **11**: 15–20

© 1999 Cancer Research Campaign
